# A Designed Analog of an Antimicrobial Peptide, Crabrolin, Exhibits Enhanced Anti-Proliferative and In Vivo Antimicrobial Activity

**DOI:** 10.3390/ijms241914472

**Published:** 2023-09-23

**Authors:** Aifang Yao, Yingxue Ma, Ruize Sun, Wanchen Zou, Xiaoling Chen, Mei Zhou, Chengbang Ma, Tianbao Chen, Chris Shaw, Lei Wang

**Affiliations:** 1 College of Biological Science and Engineering, Fuzhou University, Fuzhou 350108, China; 2 School of Pharmacy, Queen’s University Belfast, 97 Lisburn Road, Belfast BT9 7BL, UK; myx951223@163.com (Y.M.); rsun03@qub.ac.uk (R.S.); x.chen@qub.ac.uk (X.C.); m.zhou@qub.ac.uk (M.Z.); c.ma@qub.ac.uk (C.M.); t.chen@qub.ac.uk (T.C.); chris.shaw@qub.ac.uk (C.S.); l.wang@qub.ac.uk (L.W.)

**Keywords:** antimicrobial peptides, anti-infective materials, bacterial biofilms, cancer, crabrolin, *Vespa crabro*, *Galleria mellonella*, peptide design

## Abstract

Antimicrobial peptides have gradually attracted interest as promising alternatives to conventional agents to control the worldwide health threats posed by antibiotic resistance and cancer. Crabrolin is a tridecapeptide extracted from the venom of the European hornet (*Vespa crabro*). Its antibacterial and anticancer potentials have been underrated compared to other peptides discovered from natural resources. Herein, a series of analogs were designed based on the template sequence of crabrolin to study its structure–activity relationship and enhance the drug’s potential by changing the number, type, and distribution of charged residues. The cationicity-enhanced derivatives were shown to have improved antibacterial and anticancer activities with a lower toxicity. Notably, the double-arginine-modified product, crabrolin-TR, possessed a potent capacity against *Pseudomonas aeruginosa* (minimum inhibitory concentration (MIC) = 4 μM), which was around thirty times stronger than the parent peptide (MIC = 128 μM). Furthermore, crabrolin-TR showed an in vivo treatment efficacy in a *Klebsiella-pneumoniae*-infected waxworm model and was non-toxic under its maximum MBC value (MIC = 8 μM), indicating its therapeutic potency and better selectivity. Overall, we rationally designed functional peptides by progressively increasing the number and distribution of charged residues, demonstrating new insights for developing therapeutic molecules from natural resources with enhanced properties, and proposed crabrolin-TR as an appealing antibacterial and anticancer agent candidate for development.

## 1. Introduction

ESKAPE pathogens (*Enterococcus faecium* (*E. faecium*), *Staphylococcus aureus* (*S. aureus*), *Klebsiella pneumoniae* (*K. pneumoniae*), *Acinetobacter baumannii*, *Pseudomonas aeruginosa* (*P. aeruginosa*), and *Enterobacter species*), which include six nosocomial pathogens, have caused a highly complex and concerning situation for human beings due to their growing multi-drug resistance and virulence [1]. Their ability to cause post-translational self-modifications and biofilm-mediated hospital infections makes ESKAPE pathogens ‘escape’ the biocidal action of almost all approved antibacterial agents on the market [2,3,4,5,6]. Therefore, developing new antibiotics to treat drug-resistant infections, especially those caused by ESKAPE pathogens, is a pressing matter at the moment. On the other hand, some studies have confirmed that bacterial infections may have a non-negligible relationship with the occurrence of some cancers [7]. For example, Helicobacter pylori (*H. pylori*) is a spiral-shaped bacterium, and it has caused about 810,000 infections worldwide, which is the most powerful evidence that illustrates that there is a link between microorganisms and cancer development [8]. For a better understanding of the role of bacteria in the development of cancer, efforts have also been made by scientists to explore the tumor cell microenvironment. Recently, researchers from the Weizmann Institute of Science undertook as study of a 16S ribosomal DNA (rDNA) analysis method to identify and characterize all the bacteria in tumor samples. They found that the cancer cells showed a stronger proliferation and migration capacity in bacteria-enriched areas, somehow helping tumor cells to evade the immune response or even preventing them from being destroyed by chemotherapeutic drugs [9]. This suggests the feasibility of treating or preventing cancers by developing drugs that target tumor-associated microbes. Accordingly, a study published in the Journal of Cell Report indicated that the efficacy of the chemotherapeutic agent 5-fluorouracil in the treatment of colorectal cancer could be attributed to its unexpectedly potent killing capacity towards intratumoral microbiota [10]. These studies have attracted the attention of researchers to discover drugs with broad-spectrum antibacterial and anticancer dual effects.

Antimicrobial peptides (AMPs), in general, are considered to be short peptides that are positively charged and amphiphilic [11]. Due to their excellent pharmacological functions that have been continuously discovered, they have gradually attracted the interest of researchers from all over the world. The rapid bactericidal activity of AMPs makes them promising candidates for therapeutic anti-infectives. Daptomycin is a cyclic AMP approved by the FDA in 2003 and was recently introduced into clinical practice for the treatment of complicated skin and skin-structure infections (cSSSI) caused by Gram-positive bacteria, especially *S. aureus* [12]. The application of AMPs in dentistry, the treatment of surgical infection, wound healing, and ophthalmology is continuously developing. For instance, ZXR-2 (FKIGGFIKKLWRSLLA) possesses an excellent activity against *Porphyromonas gingivalis*, *Streptococcus sobrinus*, and *Streptococcus mutans*, and it could be used to treat dental caries and dental pulp infections [13]. Meanwhile, studies have shown that AMP PXL150 has a significant effect on burn wound infections [14]. Moreover, the use of these drugs is only in the theoretical stage but is showing good application prospects already. Specifically, pexiganan, a derivative of magainin with 22 amino acids, formulated as a topical cream, is in a phase III clinical trial to treat diabetic foot ulcers caused by bacterial infections (clinical trial numbers: NCT00563394 and NCT00563433) [15].

There are numerous resources that can be used to discover AMPs [16]. Due to its critical role in hunting and resisting the invasion of predators, the venom of various insects and amphibians has become a treasure trove for discovering AMPs [17]. Among them is wasp venom [18]. As early as 1983, researchers have extracted AMPs from wasp venoms, such as mastoparan and crabrolin, but compared to other insect peptides that have been widely studied, the potential of this natural resource remains underrated [19]. Two years ago, our team improved the biological activity and enhanced the stability of mastoparan through a target design, which proved the development potential of wasp venom [20].

Since crabrolin was first isolated and identified from the European hornet, *Vespa crabro*, in 1983, its potential drug value has increasingly been demonstrated [19]. By 1997, crabrolin was accurately proven to have antibacterial potential. In 1999, Krishnakumari and other scientists discovered that crabrolin possesses an LPS-neutralizing ability and may have potential anti-inflammatory activity [21]. In 2017, the secondary structure of crabrolin was comprehensively studied and identified, and its α-helix conformation was finally determined. Subsequent research in 2020 provided further insights into its functionality [22,23,24]. In addition, as previously reported, researchers have characterized the structure and investigated the biological activity of some crabrolin peptide isoforms [23,25]. Among those designed peptides, its cationicity-enhanced analog, crabrolin Plus, displayed an improved antibacterial activity [23]. Furthermore, strategies have been continually explored by scientists to modify the functional properties of crabrolin derivatives. Accordingly, a newly designed peptide, crabrolin21, obtained by artificially adding eight amino acid fragments, which comprised two positively charged residues alternated by hydrophobic residues on the C-terminus of crabrolin Plus, exhibited a higher inhibiting capacity and selectivity towards bacteria [25]. Crabrolin has the potential to be developed into an antibacterial drug. These studies also pointed out some limitations in the development of crabrolin, such as its unremarkable antibacterial property, single effects, and a lack of toxicity studies.

In this study, crabrolin was used as a template and modified according to its amino acid sequence, including changing the side chain group of the charged amino acid and the position and quantity of charged amino acids. Finally, we obtained seven modified crabrolin derivatives and evaluated their secondary structure, toxicity, antibacterial activity, and anti-proliferative activity on cancer cells. In addition, we also briefly discussed the possible mechanism of crabrolin and its derivatives by analyzing the cell membrane permeability of bacteria and cancer cells. On the one hand, we attempted to further explore the effects of the amount, type, and concentration of charged amino acids on crabrolin’s biological activity; on the other hand, we expected to find a potential drug candidate from the modification products of crabrolin as well as its possible mechanism to treat bacteria and cancer cells.

## 2. Results

### 2.1. Design of Crabrolin Derivatives

**Figure 1:**
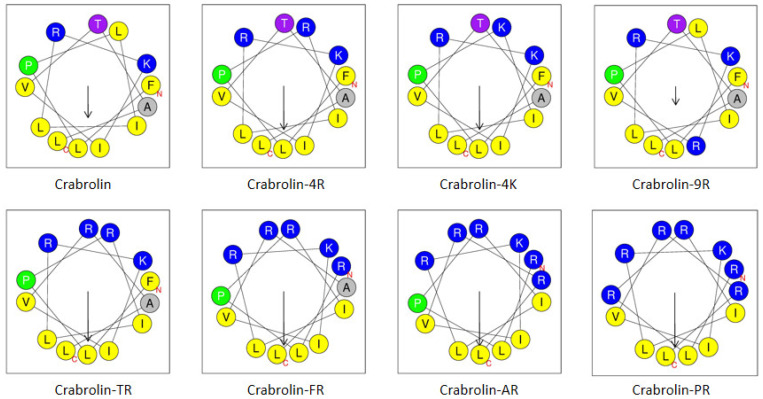
Helical wheel projections of crabrolin and its derivatives. The arrows indicate the direction of summed vectors of hydrophobicity. Amino acids in blue color are positively charged, and amino acids in yellow color are hydrophobic. The red letters C and N indicate the C-and N-terminals, respectively.

To study the structure–activity relationship of crabrolin, seven derivatives with different charges and amphipathic properties were designed and tested for their antibacterial activities. Here, seven derivatives were designed by changing the number, the type, and the distribution of the charged amino acids to study how the charged amino acids influence the secondary structure and bioactivity of crabrolin (Table 1 and Figure 1). Crabrolin is composed of thirteen amino acids (including two positively charged amino acids), and there is a natural amidation modification at its C-terminus. Through the calculation of its hydrophobicity by the Heliquest server, the hydrophobicity of crabrolin was found to be 0.977 (the highest of all the tested peptides).

The purpose of designing crabrolin-4R and crabrolin-4K was to understand the effect of different types of positively charged amino acids on the biological activity of crabrolin. Here, the fourth position of leucine was replaced by arginine and lysine to obtain crabrolin-4R and crabrolin-4K, respectively. Crabrolin-4R and crabrolin-4K had the same number of charges, but the hydrophobicity of crabrolin-4K was slightly higher than that of crabrolin-4R.

The purpose of designing crabrolin-9R was to compare the effect of the position of the charges on the activity of the AMPs by comparing it with that of crabrolin-4R. Compared with crabrolin-4R, which had a concentrated charged surface, the distribution of the charged amino acids of crabrolin-9R was dispersed, and the hydrophobic face of crabrolin-9R was broken. Crabrolin-9R had a similar hydrophobicity and shared the same molecular mass and charge number as crabrolin-4R. Crabrolin-9R was designed to have a relatively dispersed charge distribution by replacing the leucine in the ninth position of crabrolin with arginine.

To study the effect of the number of charges on the activity of crabrolin, crabrolin-TR, crabrolin-FR, crabrolin-AR, and crabrolin-PR were designed to gradually add charged amino acids to the hydrophilic face to form a more obvious hydrophilic surface and finally obtain a “perfect conformation” (crabrolin-PR) through the prediction of helical wheel projections. With the addition of the charged amino acids, the number of charges on these derivatives increased (the number of charges of crabrolin-PR was the highest among all the peptides tested), and the hydrophobicity decreased (where crabrolin-PR had the lowest hydrophobicity of all the peptides tested).

### 2.2. The Secondary Structure of Crabrolin and Its Derivatives

**Figure 2:**
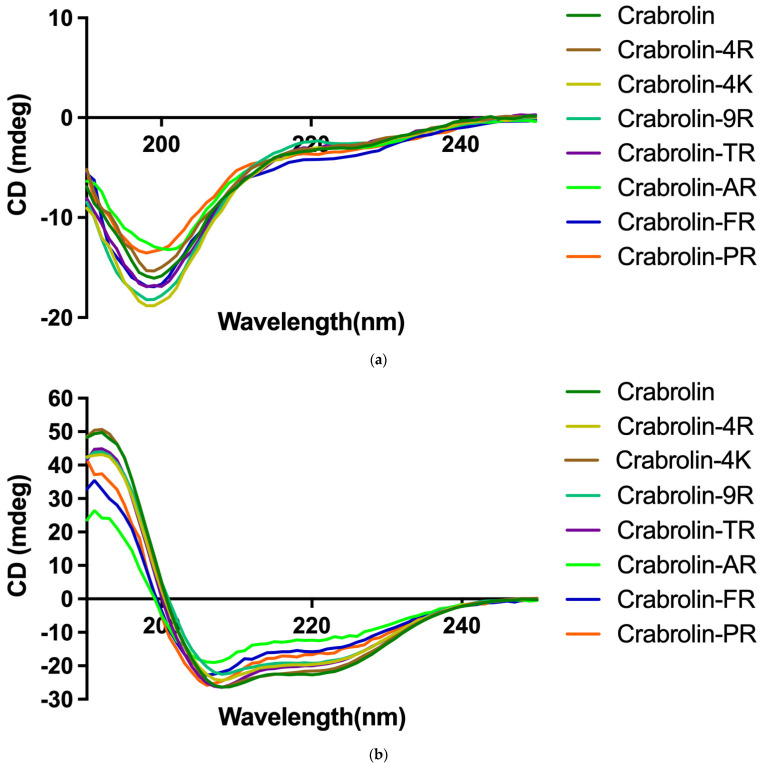
Secondary structure of crabrolin and its derivatives analyzed by Circular Dichorism Spectroscopy in (**a**) 10 mM ammonium acetate (NH_4_AC) and (**b**) 50% trifluoroethanol (TFE)/10 mM NH_4_AC. The spectra were averaged over three consecutive scans, and the solvent CD signal was subtracted.

To better study the secondary structure of crabrolin and its derivatives, the Circular Dichorism (CD) spectrum was used. The crabrolin and its derivatives all adopted a random coil structure in a 10 mM ammonium acetate (NH_4_AC) environment, as evidenced by a distinct negative peak at 200 nm (Figure 2a). However, all these tested peptides showed negative peaks at 222 nm and 208 nm in a 50% trifluoroethanol (TFE)/10 mM NH_4_AC environment (Figure 2b), which indicated that, in this membrane-mimicking environment, the crabrolin and its derivatives adopted α-helix conformations.

### 2.3. Antibacterial Activity of Crabrolin and Its Derivatives

By measuring the minimum inhibitory concentration (MIC) and minimum bactericidal concentration (MBC) values of crabrolin and its derivatives, it was possible to further compare the differences in their antibacterial activities to understand the structure–activity relationships of crabrolin and its derivatives. It was found from the data shown in Table 2 that crabrolin showed a moderate antibacterial effect on Gram-negative bacteria but a strong antibacterial effect on Gram-positive bacteria, especially for *S. aureus* (MIC *=* 2 μM). Compared with the parent peptide, crabrolin, crabrolin-4R and crabrolin-4K showed a significantly improved antibacterial activity (eight times higher) on Gram-negative bacteria, and in terms of their abilities to treat Gram-positive bacteria, that of crabrolin-4R remained the same as crabrolin, while that of crabrolin-4K slightly increased. Obviously, crabrolin-9R with its three dispersed charges had a greatly reduced antibacterial activity. As the number of the charged residues increased, the antibacterial activity towards bacteria increased and finally remained unchanged or even slightly decreased. Among the designed peptides, crabrolin-TR showed the best antibacterial effects, with MIC values ranging from 1 μM to 8 μM. Moreover, the antibacterial activity of crabrolin-TR towards *P. aeruginosa* was 32-fold higher than that of the parent peptide.

### 2.4. Antibiofilm Activity of Crabrolin and Its Derivatives

To explore the effect of crabrolin and its derivatives against drug-resistant biofilm-forming strains, the MBIC (minimum biofilm inhibitory concentration) and MBEC (minimum biofilm eradication concentration) values were measured. As shown in Table 3, the antibiofilm effect of crabrolin against biofilms of Gram-positive bacteria was stronger than it was against biofilms of Gram-positive bacteria. Specifically, crabrolin had a significant biofilm-inhibiting effect on methicillin-resistant *Staphylococcus aureus* (MRSA) and could also eradicate this biofilm at large concentrations. Compared with the parent peptide, the cationicity-enhanced analogs crabrolin-4R and crabrolin-4K showed greatly increased biofilm-inhibiting abilities against Gram-negative bacteria. As for crabrolin-9R, which did not have a concentrated hydrophilic surface, it had no inhibiting or eradication effect on the biofilms of the tested resistant bacteria. Similar to the antibacterial experiment results, crabrolin-TR exhibited the strongest inhibitory effect on biofilm growth, especially on the MRSA biofilm (MBIC *=* 2 µM). Compared with their abilities to inhibit the growth of the biofilms, their abilities to eradicate the biofilms were greatly reduced. As the number of charges continued to increase (crabrolin-FR, crabrolin-AR, and crabrolin-PR), their effects on the Gram-positive bacterial biofilm decreased, but their inhibitory effect on the Gram-negative bacterial biofilms remained unchanged.

### 2.5. Anti-Proliferation Activity of Crabrolin and Its Derivatives

**Figure 3:**
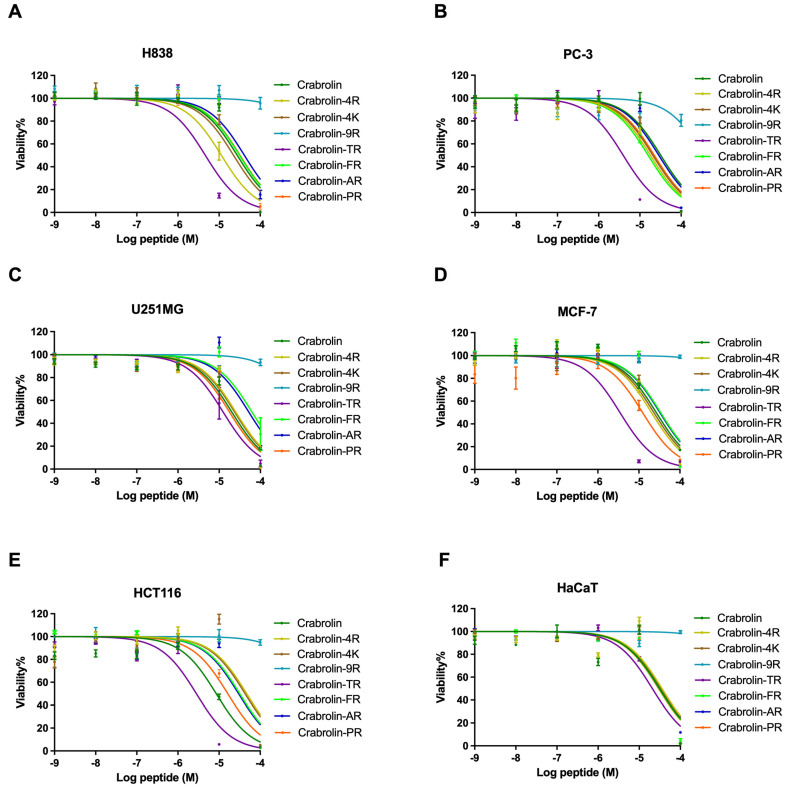
The dose–response curves showing half-maximal inhibitory concentration (IC_50_) values of crabrolin and its derivatives for inhibition of (**A**) human non-small-cell lung cancer (H838) cell line, (**B**) human prostate carcinoma (PC-3) cell line, (**C**) human glioblastoma astrocytoma (U251MG) cell line, (**D**) human breast cancer (MCF-7) cell line, (**E**) human colorectal carcinoma (HCT 116) cell line, and (**F**) human keratinocyte (HaCaT) cell line viability. The percentage was calculated based on the effect induced by a positive control, i.e., 1% Triton X-100. Treatment with PBS was used as a negative control. The error bars in the graph around mean data points indicate the standard error of the mean (SEM).

To investigate the anticancer effects of crabrolin and its derivatives and the relationship between their structure and anticancer efficacy, MTT assays were used to detect the anti-proliferative effects of these peptides. Crabrolin and its analogs, except for crabolin-9R, all exhibited broad-spectral anti-proliferation activities against the tested human cancer cells (Figure 3). As summarized in Table 4, crabrolin had the most potent anti-proliferative effect against the human colorectal carcinoma (HCT-116) cells (half-maximal inhibitory concentration (IC_50_) reached 8.529 µM) and was less effective against the human prostate carcinoma (PC-3) cells (IC_50_ reached 32.13 µM). The anti-proliferation effect of crabrolin against the human keratinocyte cell line (HaCaT) was weaker than that against the PC-3 cell line, which showed the selectivity of crabrolin. Compared with the parent peptide, the modified products, crabrolin-4K and crabrolin-4R, had weakened activities against the human glioblastoma astrocytoma (U251MG) and HCT-116 cell lines, but it showed enhanced anti-proliferation effects against the other cell lines. In addition, compared with crabrolin-4K, crabrolin-4R had significantly increased anti-proliferative effects against the human non-small-cell lung cancer line H838 and the human prostate carcinoma PC-3 cells. This marked increase, however, was not observed for the other cell lines. For the normal cells, the cytotoxicity of crabrolin-4R and crabrolin-4K against the HaCaT cell line was not much different from that of the parent peptide. As for crabrolin-9R, it did not show obvious anti-proliferation effects against the six cell lines tested. Notably, crabrolin-TR showed the most significant anti-proliferation effect against the tested cancer cell lines among the crabrolin and its derivatives. For the five cancer cell lines tested, the IC_50_ values were all less than 10 µM except for the U251MG cells, whose IC_50_ was greater than 10 μM. The IC_50_ value against the HCT-116 cells was the lowest, reaching 2.810 μM. Crabrolin-FR, crabrolin-AR, and crabrolin-PR with their increased number of charges, had lower anti-proliferative activities than crabrolin-TR, and the anti-proliferative activity of crabrolin-PR was relatively high (for example, for the human breast cancer (MCF-7) cells, its IC_50_ reached 12.29 µM). The anti-proliferation effects of these three derivatives on the normal cells were similar.

### 2.6. The Cytotoxicity of Crabrolin and Its Derivatives

**Figure 4:**
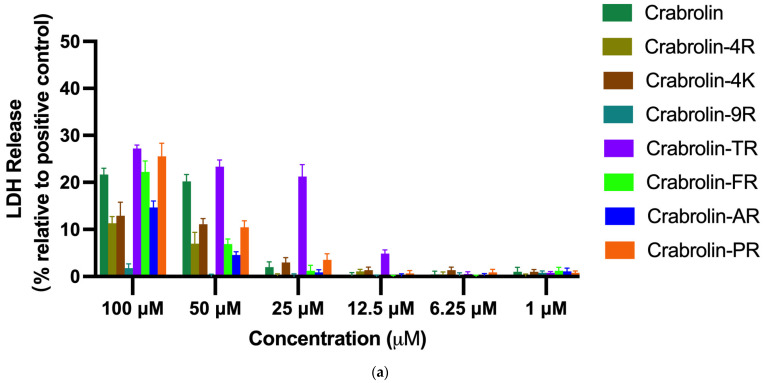

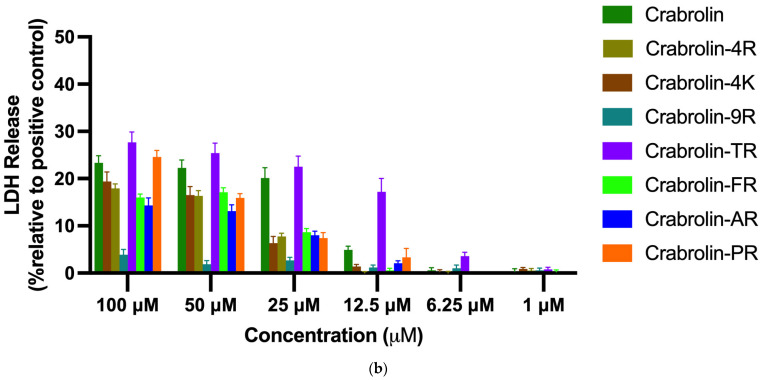
The release of lactate dehydrogenase (LDH) from (**a**) the immortalized human microvascular endothelial cell line HMEC-1 cells and (**b**) HaCaT cells incubated in the presence of crabrolin and its derivatives. The percentage was calculated based on the effect induced by a positive control, i.e., 1% Triton X-100. Treatment with PBS was used as a negative control.

Through a lactate dehydrogenase (LDH) experiment, the cytotoxicity of crabrolin and its derivatives was determined for normal cells using the human keratinocyte cell line HaCaT and the immortalized human microvascular endothelial cell line HMEC-1. Overall, at a concentration of 100 μM, all the tested peptides, except crabrolin-9R, were cytotoxic to the normal cell lines. A similar percentage of LDH release was observed after a 50 μM exposure to the peptides. Specifically, crabrolin-TR, enriched with four positive charges on the hydrophilic face, caused the highest LDH release among these peptides. In contrast, crabrolin-9R, which had a dispersed charged distribution, showed the lowest cytotoxicity. At a concentration of 25 μM, for the HMEC-1 cell line, only crabrolin-TR was cytotoxic, while the other peptides were not. For the HaCaT cell line, crabrolin-TR and the parent peptide induced about a 20% LDH leakage, while the other tested peptides were not that cytotoxic with LDH release levels below 10%. As shown in Figure 4, none of the tested peptides produced a significant cytotoxicity on the normal cell lines under a 6.25 μM treatment.

### 2.7. Hemolysis of Crabrolin and Its Derivatives

**Figure 5:**
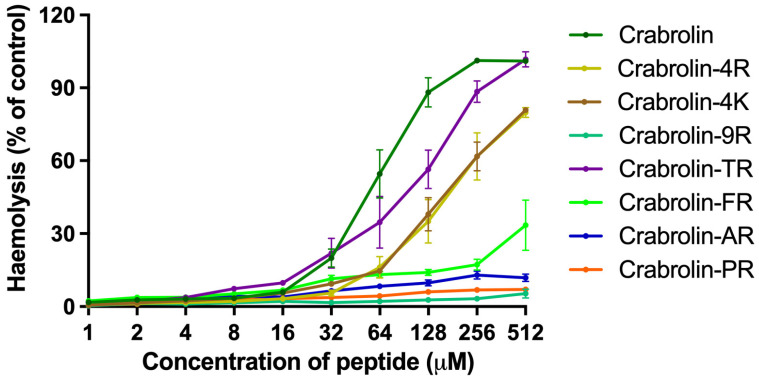
The hemolytic activities of crabrolin and its derivatives at concentrations ranging from 1 to 512 μM. The percentage was calculated based on the effect induced by a positive control, i.e., 1% Triton X-100. Treatment with PBS was used as a negative control. Error bars indicate standard deviation (SD) of fifteen replicates in three tests (five replicates each time).

As shown in Table 5 and Figure 5, the hemolytic activities of the modification products of crabrolin were reduced. As the number of charged residues increased, the hemolytic abilities of the antibacterial peptides gradually decreased. Among them, crabrolin-PR had the lowest hemolytic activity (less than 1% at the measured maximum concentration). For crabrolin-4R and crabrolin-4K, which had different charged amino acids, their hemolytic activity curves overlapped, which means that their abilities to destroy the erythrocytes were equivalent. Crabrolin-9R, which had a dispersed distribution, lost its hemolytic activity. In addition, by comparing their hemolytic abilities under the maximum MBC, crabrolin-TR and crabrolin-PR showed better selectivities (less than 5% hemolytic activity under the maximum MBC concentration).

### 2.8. Membrane Permeability Activity against H838 Cell Line and Escherichia coli (E. coli)

**Figure 6:**
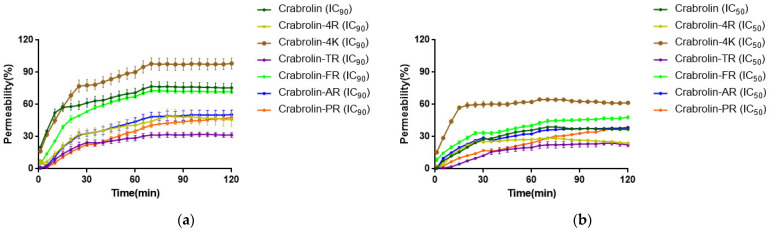
Kinetics of membrane permeabilization of crabrolin and its derivatives on H838 cell line at (**a**) IC_90_ concentration and (**b**) IC_50_ concentration. The percentage of membrane permeabilization was measured using bacterial cells treated with 70% isopropanol.

To explore the mechanism of action of crabrolin and its derivatives and compare their killing speeds, cell permeability assays were performed using Sytox Green assays. The dynamic permeability curve of crabrolin and its derivatives on the H838 cell line showed that the permeability rate of all the samples at the IC_90_ concentration (Figure 6a) was higher than that at the IC_50_ concentration (Figure 6b), and the permeability rate continued to increase with time and finally remained constant. It was found that crabrolin could cause about an 80% cell membrane destruction at its IC_90_ concentration in 2 h, which indicated that the destruction of the cell membrane was the main mechanism leading to the anti-proliferation effect of crabrolin on the H838 cell line. With the increase in the number of charges (crabrolin, crabrolin-4R, and crabrolin-TR), the permeability rate of the cancer cells gradually decreased. When the number of net charges reached four (crabrolin-TR), the permeability rate dropped to the lowest. Then, when the number of charged amino acids continued to increase, the membrane permeability rate increased sharply (crabrolin-FR) and then continued to decrease (crabrolin-AR and crabrolin-PR).

**Figure 7:**
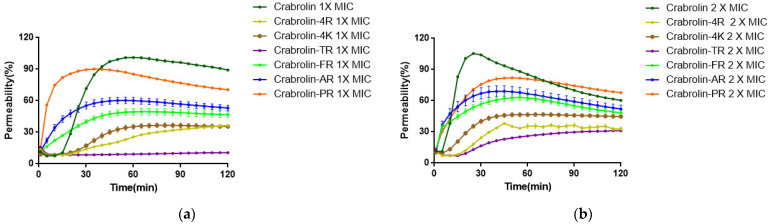
Kinetics of membrane permeabilization of crabrolin and its derivatives on *Escherichia coli* (*E. coli*) (ATCC 8739) at (**a**) 1 × MIC and (**b**) 2 × MIC. The percentage of membrane permeabilization was measured using bacterial cells treated with 70% isopropanol.

For crabrolin, both the MIC (Figure 7a) and the 2 × MIC (Figure 7b) concentrations reached about a 100% lysis rate in a short time, which proved that destroying the cell membrane was one of its antibacterial mechanisms. At the same time, crabrolin-4K and crabrolin-4R had low membrane lysis rates at both concentrations, and compared to crabrolin-4K, crabrolin-4R’s membrane lysis rate was lower. With the increase in the number of charges, the membrane permeability rate reached the minimum (crabrolin-TR), and the cell membrane of *E. coli* was not ruptured at the 1 × MIC concentration, but as the number of charges continued to increase, the membrane permeability rate gradually increased until crabrolin-PR reached the highest (basically consistent with the parent peptide).

### 2.9. Crabrolin-TR and Crabrolin in the Treatment of Larvae Infected with K. pneumoniae (ATCC 43816)

**Figure 8:**
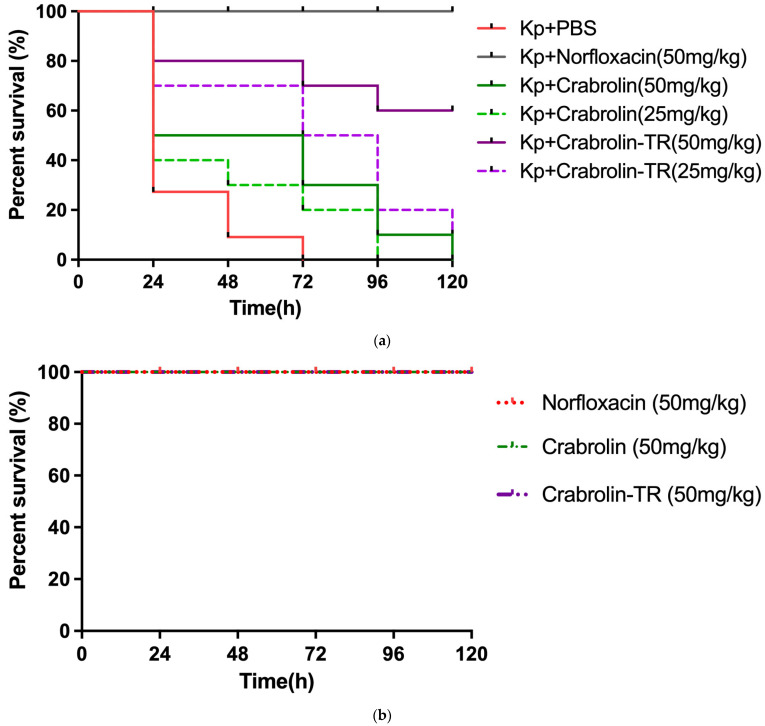
(**a**) The mortality of *Galleria mellonella* larvae infected with *K. pneumoniae* (ATCC 43816) treated with crabrolin-TR (50 mg/kg, 25 mg/kg) and crabrolin (50 mg/kg, 25 mg/kg). Infected larvae treated with 20 mg/kg of norfloxacin were regarded as a positive control. Infected larvae treated with PBS were regarded as a negative control. (**b**) Larvae without infection were treated with 50 mg/kg crabrolin and crabrolin-TR, which were applied to assess their potential toxicities to the hosts.

To better study their antibacterial effects in vivo, an infected *Galleria mellonella* larvae model was used to test and compare the antibacterial effects of crabrolin and its derivative, crabrolin-TR, with their in vitro antibacterial abilities. As shown in Figure 8a, after the treatment with crabrolin-TR, the mortality of the *K. pneumoniae* infection was significantly reduced. Also, the highest doses of crabrolin-TR and crabrolin did not result in the death of healthy larvae (Figure 8b). Compared with crabrolin, crabrolin-TR was more effective in treating the larvae. At higher concentrations (50 mg/kg), crabrolin-TR had a five-day survival rate for the larvae of 50%. At lower concentrations (25 mg/kg), the crabrolin-TR-treated larvae had a five-day survival rate of only 10%.

## 3. Discussion

During the process of animal evolution, some creatures have gradually developed technical organs to produce and inject venom, and different species have various venom components [26]. Recent studies have shown that a single type of venom can contain hundreds of individual toxins, mainly proteins and peptides, which produce a variety of physiological effects [27]. On the one hand, precisely because of the complexity of animal venom, it has become a treasure trove for the discovery of new drugs [28]. On the other hand, minor changes in the amino acid sequence of these isolated components usually give rise to updated performances in terms of their biological properties, leading to the appearance of new toxins [16]. Therefore, drug modifications based on the amino acid sequence of known toxins as prodrugs has become another crucial direction for the development of new drugs derived from animal venom [29]. Among these, peptides extracted from wasp venom, for example, crabrolin, have the potential to be used as precursors for drug development due to their anti-infective biological activity, which was discovered in previous studies [19,20,21,22,23]. However, the development potential of crabrolin has previously been underestimated compared to other well-studied AMPs extracted from wasps, such as mastoparan [30,31]. This was possibly due to its inconspicuous anti-infective activity and uncertain toxicity.

In this study, we reassessed the antibacterial effects, anti-proliferation properties, and toxicity of crabrolin. The experimental results showed that for the six tested bacteria, crabrolin had a potent antibacterial effect on the Gram-positive bacteria. Among them, the MIC values for the two multi-drug-resistant bacteria, MRSA and *E. faecium*, reached 8 and 16 µM, respectively. However, for the Gram-negative bacteria, the antibacterial effect was not marked. This may be attributed to the low affinity between crabrolin and the negatively charged substances on the membranes of Gram-negative bacteria, such as LPS, thereby limiting its ability to penetrate the outer membrane of the bacteria. Analogously, the experiments on the inhibition and eradication of biofilms also illustrated the same situation. Crabrolin tended to inhibit the production of Gram-positive bacterial biofilms, and it was difficult for the peptide to eradicate the biofilm once formed, which may be related to the composition of the biofilm itself [32]. Biofilms are composed of polysaccharides, DNA/RNA, proteins, and water. Once these components are formed, it is difficult to completely eradicate them [33]. In the anti-proliferation experiment, crabrolin showed a moderate anticancer activity using six types of cancer cell lines. Among these, crabrolin had the best anti-proliferation effect against the HCT-116 cell line, with an IC_50_ value of 8.539 µM. In addition, we also evaluated the cytotoxicity of crabrolin towards normal cells and its hemolytic activity. The experimental results indicated that crabrolin showed cytotoxicity towards the normal cells (HMEC-1 and Hacat cell lines) at the largest test concentration (100 µM), and its hemolytic activity was also high (100% at 256 µM). In summary, crabrolin has a good antibacterial effect against Gram-positive bacteria, but its biological activity against Gram-negative bacteria and cancer cells is not outstanding, and it has a certain degree of toxicity.

In order to study the effects of different charged amino acids on the biological activity of crabrolin, we replaced the leucine in the fourth position of crabrolin with arginine and lysine to obtain crabrolin-4R and crabrolin-4K, respectively. Compared with crabrolin-4K, crabrolin-4R showed better effects in the antibacterial and anti-proliferation experiments. In addition, the hemolysis tests on horse blood cells and the LDH release toxicity tests on the HMEC-1 and HaCaT cell lines proved that the crabrolin-4R and crabrolin-4K basically had the same hemolytic activity and cytotoxicity. In the Sytox Green permeability assay, we found that for both the H838 cell line and the *E. coli* bacteria, crabrolin-4K had a higher membrane permeability rate than crabrolin-4R, which may have been due to the different modes of action between the two analogs on the cell membrane [34], or the existence of other antibacterial and anticancer mechanisms may have made the membrane permeability rate of the two deviate from the results of the activity test [35], a thought which requires further study and verification. Previous studies have shown that lysine and arginine have the same number of positive charges and similar chemical properties, suggesting their similar role in the amino acid sequence, but as our experimental results and many studies have confirmed, their effects appear to be different [36,37]. Compared to lysine, the guanidine group of arginine allows for interactions in three possible directions, allowing arginine to form stronger electrostatic interactions [38,39]. Also, the higher pKa of the guanidine group of arginine may be more stable than the ionic interaction caused by the amino group of lysine [38]. These data can explain why arginine is better than lysine in AMP design. One study replaced lysine in the sequence of LL-37, which was derived from humans with arginine [40]. The results showed that arginine plays an important role in the anti-infective activity of AMPs. Also, replacing the lysine in an antimicrobial peptide sequence extracted from horseshoe crab hemocytes with arginine resulted in a modified product with a better activity and a lower toxicity than the original peptide [41].

To study the effect of the charge density on the biological activity of crabrolin, we designed crabrolin-9R with dispersed charged amino acids, and this was compared with crabrolin-4R, in which the charged amino acids were clustered. Apparently, crabrolin-9R was almost devoid of all the biological activities tested and only had a weak effect on *S. aureus* (NCTC 10788) and *E. coil* (ATCC-8739). However, it also lost its toxicity and hemolytic activity against normal human cells and horse erythrocytes. These data indicated that the charge density may be a crucial factor in the biological activity of AMPs. In a previous study, some researchers found that when an antibacterial peptide is folded into a tighter conformation and the net charge density increases, the antibacterial effect is greatly improved [42].

Similarly, in this study, based on crabrolin, we obtained crabrolin-4R, crabrolin-TR crabrolin-FR, crabrolin-AR, and crabrolin-PR by replacing non-charged hydrophilic amino acids or hydrophobic amino acids with arginine with a positive charge in order to investigate the role of the number of charges on the bioactivity of crabrolin. In terms of the antibacterial activity, as the number of net charges increased, the antibacterial activity was significantly improved, and when the number reached four (crabrolin-TR), it reached a peak and then remained unchanged. In terms of the anti-proliferation activity, similarly, when the cumulative number of charged amino acids reached four (crabrolin-TR), the anticancer activity reached a maximum, and then, as the number of net charges continued to increase, the anti-proliferation effect against the three tested cancer cell lines decreased, which was different to the performance of these derivatives against bacteria. These results indicated that there exists an ideal ratio of positively charged amino acids to hydrophobic amino acids. Once this ratio was reached, the biological activity of the peptide reached its maximum value. Anything greater or less than this value reduced its biological activity, and the difference in the antibacterial activity and anti-infective activity can be explained by the different compositions of the bacterial membranes and cancer cell membranes [43,44]. Jiang et al. studied the relationship between the net charges and biological activity of L-V13K and proved that charged amino acids have a significant effect on the hemolysis and antibacterial properties of AMPs [45]. The team of Ma studied leucocin A, an AMP extracted from *Leuconostoc gelidum*, and found that only a certain appropriate charge-to-hydrophobicity ratio could make the AMP reach the maximum biological activity [46]. Similarly, researchers believe that the activity of AMPs increases with the increase in the level of positive charge on the peptide. Conversely, when the charge increases to reach a critical value, the opposite behavior is observed, beyond which the activity decreases [47].

The cytotoxic effects of crabrolin and its designed analogs in normal cell lines were also confirmed by LDH assays. LDH is an intracytoplasmic enzyme released into the culture medium when the cell membrane is damaged [48]. To some extent, the enzyme activity in the culture medium is proportional to the number of lysed cells. Therefore, we assessed the extent of damage to normal cells caused by crabrolin and its analogs by detecting the release of LDH. After the peptide treatment, the level of LDH release by the HMEC-1 and HaCaT cells was revealed to be concentration-dependent. In addition, no cytotoxicity was detected at concentrations below 6.25 μM, where crabrolin-TR was active against most of the resistant bacteria and cancer cells, indicating its safety. We also observed a minimal LDH leakage after exposure to crabrolin and its designed analogs at the maximum concentration (100 μM). Combined with the experimental results of the membrane permeability test, it is reasonable to speculate that crabrolin and its analogs exert bacteriostatic and anti-proliferative capacities through the membranolytic and non-membranolytic pathways. Similarly, a previous study has reported that D-LAK inhibits cancer cell proliferation, resulting in LDH release, cell cycle arrest, ROS production, and mitochondria-mediated apoptosis via both membrane damage and non-membrane lysis pathways [49].

Among all the designed derivatives of crabrolin, crabrolin-TR displayed a significant enhancement in terms of its antibacterial effect against bacteria, especially Gram-negative bacteria, without exhibiting a significant toxicity under the effective concentration. Additionally, compared to previously reported crabrolin analogs, our designed peptide, crabrolin-TR, showed a more potent antibacterial activity with a lower cytotoxicity. For example, crabrolin Plus could inhibit the growth of *E. coli* at a concentration of 24 μM, while the MIC of crabrolin-TR against *E. coli* was 2 μM, which was two times stronger than that of crabrolin21 (4 μM) [23,25]. In addition, crabrolin Plus failed to exhibit any capacity against *P. aeruginosa* (>383 μM), and crabrlin21 showed efficacy at 16 μM, which was less potent than crabrolin-TR (MIC = 8 μM) [23,25]. Notably, neither crabrolin-TR nor crabrolin21 caused hemolysis at 8 μM; however, crabrolin-TR possessed a broad antibacterial activity, while crabrolin21 was only efficacious towards several bacteria at that concentration. Furthermore, when the concentration reached 100 μM, crabrolin-TR showed about a 50% hemolysis, whereas crabrolin21 resulted in an approximately 90% hemolysis of red blood cells [25]. These data indicate that crabrolin-TR is considered to become an antibacterial compound with the most potential for further research and clinical applications among the modified products of crabrolin. However, due to the single α-helix structure and the large number of charged amino acids of crabrolin-TR, protease stability may become a considerable problem. Therefore, it has the potential to be developed into a topical antibacterial drug, such as a cream or ear drop.

## 4. Materials and Methods

### 4.1. Design of Derivatives of Crabrolin

The amino acid sequence of crabrolin (FLPLILRKIVTAL-NH_2_) was obtained from the literature [19]. Seven derivatives of crabrolin were designed to study the relationship between the charge density, quantity, and charged amino acid type and its biological activity in order to look for peptides that have drug potential. First, the charged amino acids lysine and arginine with different side chains were replaced with leucine (at the 4th position) to obtain crabrolin-4K and crabrolin-4R. Then, the positions of three existing charged amino acids were changed to make them dispersed, thus obtaining crabrolin-9R. Based on crabrolin-4R, crabrolin-TR, crabrolin-FR, crabrolin-AR, and crabrolin-PR were designed, which were accompanied by an increasing number of charges.

### 4.2. Solid-Phase Peptide Synthesis and Purification

**Figure 9:**
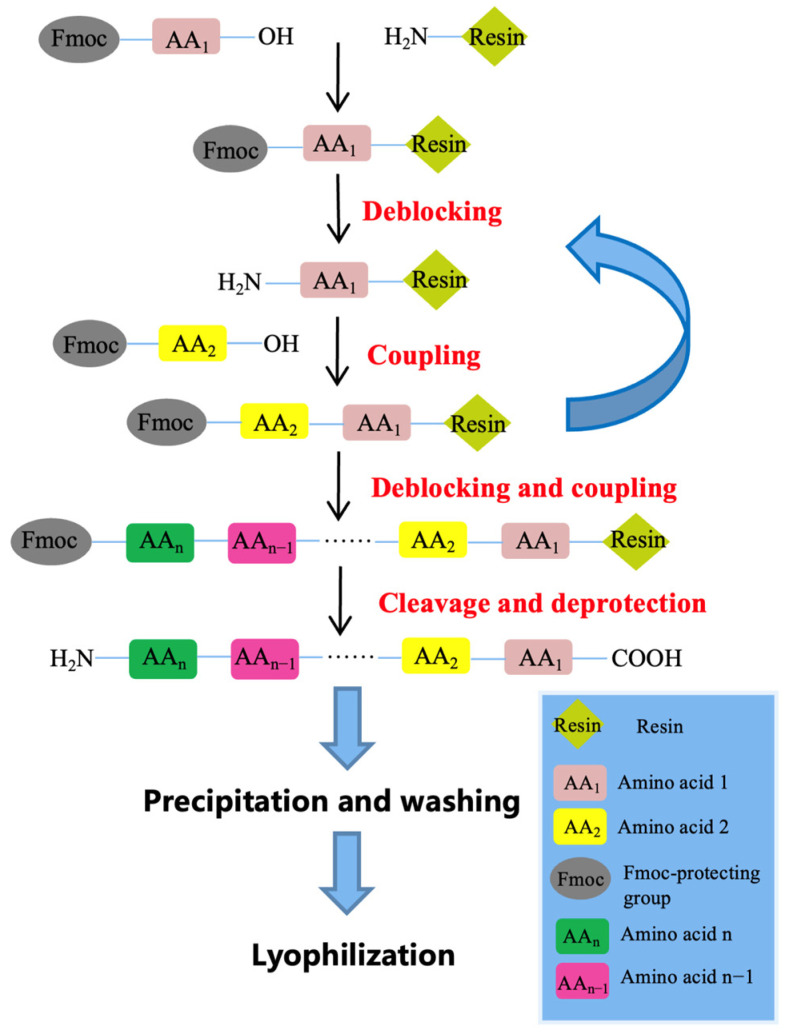
A flowchart of solid-phase peptide synthesis. Amino acids were sequentially added to the resin to build the peptide chain. The process involved a serial reaction cycle of activation, de-blocking, and coupling. The Fmoc group was deprotected from the amino acid to expose an amine and then coupled with the activated amino acid.

Crabrolin and its derivatives were synthesized by using a Tribute Peptide Synthesizer (Protein Technologies, Tucson, AZ, USA). The procedures were described in detail in a previous paper [50]. The amino acids and the activator, HBTU (2-(1H-benzotriazole-1-yl)-1,1,3,3-tetramethyluronium hexafluorophosphate), were weighed in vials. After coupling, a cleavage solution (94% trifluoroacetic acid (TFA) + 2% triisopropylsilane (TIS) + 2% 1,2-ethanedithiol (EDT) + 2% H_2_O) was prepared to perform the cleavage reaction of the protected group. Then, ice-cold diethyl ether was prepared to wash the peptide and the solution with the peptide in buffer A (TFA/water (0.05/99.95, *v*/*v*)). Finally, the crude peptide was lyophilized by a freeze dryer and then stored at −20 °C. The flowchart of peptide synthesis is shown below (Figure 9).

The crude peptide was first purified utilizing RP-HPLC. The wavelength of the detector was set at 214 nm. The RP-HPLC utilized a Cecil Adept CE4200 HPLC system (Amersham Biosciences, Buckinghamshire, UK), a Jupiter C-5 semi-preparative column (25 × 1 cm, Phenomenex, Cheshire, UK), and the Powerstream HPLC software (21 CFR part 11 compliance, Cambridge). The molecular mass of the purified peptide was obtained and confirmed by MALDI-TOF MS. First, 1 μL of the sample and 1 μL of the matrix solution (a-cyano-4-hydroxycinnamic acid (CHCA)) were spotted onto a MALDI plate at the same position. Once the samples had been air-dried, the MALDI plate was then loaded into the mass spectrometer, and the observed mass was compared with the theoretical mass values of the peptides.

### 4.3. Circular Dichorism

The secondary structure of each peptide was estimated using a CD spectrometer (Jasco J-815, Tokyo, Japan) as described in a previous study [51]. Specifically, 100 µM of each peptide, which was dissolved in 10 mM NH_4_AC buffer or 50% TFE (*v*/*v* in 10 mM NH_4_AC), respectively, was loaded into a cuvette (1 mm path length). For the analysis, three passes (accumulation) within the range of 190–260 nm were made at 20 °C and at a scanning speed of 200 nm/min, a bandwidth of 1 nm, and a data pitch of 0.5 nm.

### 4.4. Antimicrobial Susceptibility Assay

Six bacteria were tested in this experiment to detect the antimicrobial effects of crabrolin and its derivatives, including *S. aureus* (NCTC 10788), MRSA (NCTC 12493), *E. coli* (ATCC 8739), *K. pneumoniae* (ATCC 43816), *E. faecium* (NTCC-12697), and *P. aeruginosa* (ATCC 9027). The tested bacteria were cultured in Mueller Hinton broth medium (MHB) with norfloxacin (20 mg/L) as a positive control. All bacteria were incubated at 37 °C overnight and diluted to 5 × 10^5^ CFU/mL before treating with the peptides. Stock solutions of 51,200 µM peptides were prepared in phosphate-buffered saline (PBS) and further diluted by a factor of two to 100 µM. Next, 1 µL of each peptide solution was mixed with 99 µL of bacterial subculture in a 96-well plate, and the plate was incubated at 37 °C for 20 h. After culturing, the absorbance of each well in the 96-well plate was analyzed at 550 nm using a Synergy HT plate reader (BioliseBioTek, Winooski, VT, USA). The peptide concentration that resulted in no obvious bacteria in the 96-well plate was regarded as the MIC. Starting with the MIC value, 10 µL of the solution from each well was dripped onto Mueller Hinton agar (MHA) plates. The plates were cultured at 37 °C for 20 h. After culturing, the lowest concentration with no colony growth was regarded as the MBC.

### 4.5. Anti-Biofilm Assay

To measure the antibiofilm activity of crabrolin and its derivatives, four drug-resistant biofilm-forming strains, MRSA (NCTC 12493), *K. pneumoniae* (ATCC 43816), *E. faecium* (NTCC-12697), and *P. aeruginosa* (ATCC 9027), were used in this assay. Tryptic soy broth (TSB) (for the Gram-positive bacteria) and Luria–Bertani broth (LB) (for the Gram-negative bacteria) were used to culture the bacteria.

The mass of the biofilm in this assay was detected by the crystal violet staining method. First, 100 μL of 5 × 10^5^ CFU/mL bacteria was incubated with 1 μL of different peptide solutions (ranging from 51200 μM to 100 μM) for 24 h at 37 °C with 200 rpm shaking. After the biofilm formation, 100 μL of PBS was used to rinse the biofilm twice. Next, 100 µL of methanol was added to fix the biofilm, and the biofilm was then dried. Thereafter, 100 μL of 0.1% crystal violet was added and left to stain for 15 min, and then the excess crystal violet was further rinsed by PBS. After the plate had air-dried, 30% glacial acetic acid was added to each well of the plate, and the plate was shaken for 15 min before the optical density (OD) value was measured at 595 nm using a Synergy HT plate reader. The MBIC value refers to the minimum concentration at which the biofilm inhibitory rate was more than 90%.

Different from measuring the MBIC value, to determine the MBEC value, 100 μL of the bacteria was first added to 96-well plates. After the biofilm was formed, the biofilm was rinsed with PBS twice to remove the planktonic cells, and then different concentrations of the drugs were added to the biofilm (the same as the MBIC assay). The mixture was incubated at 37 °C with shaking at 200 rpm. After 24 h of incubation, the mass of the biofilm was measured in the same way as the MBIC value, and the minimum concentration at which the eradication rate was greater than 99.9% was considered to be the MBEC value.

### 4.6. Anti-Proliferative Assay

The human non-small-cell lung cancer line, H838, the human prostate carcinoma cell line, PC-3, the human glioblastoma astrocytoma, U251MG, the human breast cancer cell line, MCF-7, the human colorectal carcinoma cell line, HCT116, and the human keratinocyte cell line, HaCaT, were used to detect the anti-proliferative effects of the crabrolin and its derivatives in vitro. A total of 8000 cells were seeded in each well of a 96-well plate. After the cells had adhered to the wall, the cells were starved for 4 h. After the starvation, the cells were incubated with different concentrations of the drugs, ranging from 100 µM to 1 nM, for 24 h, with 0.1% Triton X-100 used as a positive control. Next, 10 µL of MTT (3-(4,5-dimethylthiazol-2-yl)-2,5-diphenyltetrazolium bromide) solution (5 mg/mL) was added to each well of the 96-well plate. After 2 h of incubation, 100 μL of DMSO-superseded medium was added to each well to dissolve the formazan crystals. Finally, the absorbance was detected using a Synergy HT plate reader at 570 nm.

### 4.7. LDH Cell Cytotoxicity Assay

A Pierce LDH cytotoxicity assay kit (Thermo Scientific, Waltham, MA, USA) was applied for the LDH assay. An immortalized human microvascular endothelial cell line, HMEC-1, and a human keratinocyte cell line, HaCaT, were used in the LDH assay [52]. A total of 8000 cells were placed in each well of the 96-well tissue culture plate with 100 µL of medium and incubated at 37 °C with 5% CO_2_ overnight. The cells were cultured with different drug concentrations ranging from 100 µM to 1µM for 24 h. Ten milliliters of lysis buffer was prepared as the maximum LDH activity control. After incubating at 37 °C with 5% CO_2_ for 45 min, 50 µL of the sample was transferred into each well medium in a 96-well flat-bottom plate. Then, 50 µL of the reaction mixture was transferred to the wells and mixed. The 96-well plate was cultured for 30 min and protected from light at room temperature. Then, 50 µL of stop solution (supplied in the kit) was added to each well and mixed. The absorbance was detected at 490 nm and 680 nm.

### 4.8. Hemolysis Assay

According to the procedures described in detail in a previous study, the debris and impurities of the erythrocytes were washed with PBS in advance to yield 2% of the erythrocyte suspension, and the drug concentration was diluted from 1024 µM to 2 µM with PBS [53]. The same amounts of the drug and the erythrocyte suspension were mixed and cultured at 37 °C for 2 h; 0.1% Triton X-100 (Sigma-Aldrich, St. Louis, MO, USA) was prepared as a positive control, and PBS was prepared as a negative control. After the incubation, the supernatant was transferred to a 96-well plate and then detected using a Synergy HT plate reader at 570 nm.

### 4.9. Sytox Green Permeability Assay

To explore the possible mechanism of action of crabrolin and its derivatives, the permeability of cancer cells (H838) and bacteria (*E. coli*), which had the strongest effects, were measured by a Sytox Green permeability assay [54].

To determine the permeability of the treated *E. coli*, the bacteria were cultured with LB medium to the log phase with a flowing centrifuge at 4 °C at 1000× *g* for 10 min, and then the bacteria at the bottom of the tube were collected and washed with 5% LB/0.85% NaCl solution twice. Then, the bacteria were diluted to reach the 0.7 OD value under 580 nm. Then, 40 μL of the bacteria, 10 μL of the Sytox Green, and 50 μL of the prepared drug were added to each well of a black 96-well plate. The plate was analyzed by excitation and emission wavelengths at 485 nm and 528 nm at 37 °C for 120 min (interval of 5 min) using a Synergy HT plate reader.

To determine the permeability of the treated H838 cancer cells, the H838 cells were placed on a tissue culture dish, and after the cells had adhered to the bottom of the plate, they were starved for 8 h. Then, the H838 cells were re-suspended in EBSS. After washing twice with EBSS, 80 μL of the 20,000 cell/mL H838 cells were set into each well of a black 96-well plate, and 10 μL of the Sytox Green and 10 μL of the prepared drug was added to each well of the plate. The plate was sent to the Synergy HT plate reader and analyzed in the same way as the previous description.

### 4.10. Determination of In Vivo Antimicrobial Activity of Peptides

The assessment of the in vivo antimicrobial activity of crabrolin and its derivatives was performed using the larvae of *Galleria mellonella* [55]. The infection model was constructed by injecting the larvae with 10 μL of a *K. pneumoniae* (ATCC 43816) bacteria suspension (5 × 10^4^ CFU/mL), which was prepared in PBS. After 1 h, each infected larva was further administered an injection of 10 μL of the peptide solution at different concentrations of 12.5 mg/kg, 25 mg/kg, and 50 mg/kg. Infected larvae that were administered with 10 μL of PBS were employed as a negative control, while 20 mg/kg of norfloxacin was used as a positive control. Each group contained 10 larvae, and all larvae were inspected every 24 h for 5 days.

### 4.11. Statistical Analysis

The data analysis was performed using the Prism software (Version 6.0; GraphPad Software Inc., San Diego, CA, USA). The error bars in the resulting graph indicate the standard deviation (SD) for each set of data in the 9 replicates (in 3 independent tests). One-way ANOVA analysis was applied to identify the significance by repeatedly comparing the mean value of each column with the means of the other columns (ns *p* ≥ 0.05, * *p* < 0.05, ** *p* < 0.01, *** *p* < 0.001, and **** *p* < 0.0001).

## 5. Conclusions

AMPs have the potential to be developed as therapeutic agents to combat the global health challenges of drug resistance and cancer. In this study, we employed a progressive design strategy to obtain analogs of crabrolin, whose antibacterial and anticancer effects were superior to those of the parent peptide, revealing the crucial role of the charge density and arginine in the bioactive activity of AMPs. In addition, we gradually increased the arginine residues on the hydrophilic face of α-helical AMPs and confirmed that crabrolin-TR, with its four positively charged amino acids, possessed the highest activities against the tested bacteria and cancer cells. This suggests that a balance between positively charged and hydrophobic amino acids can optimize the dual antibacterial and anticancer activities of a peptide. These findings have broadened our knowledge of the structure–activity relationship of AMPs and provided insights for developing therapeutic compounds from natural products. Furthermore, we propose that crabrolin-TR shows promise to be further investigated and developed for clinical applications to combat drug-resistant infections and cancer.

## Figures and Tables

**Table 1:** The physicochemical properties of crabrolin and its analogs. The replaced amino acid residues were highlighted in yellow.

Peptide Name	Sequence	Net Charge	Hydrophobicity <H>
Crabrolin	FLPLILRKIVTAL-NH_2_	+2	0.977
Crabrolin-4R	FLPRILRKIVTAL-NH_2_	+3	0.768
Crabrolin-4K	FLPKILRKIVTAL-NH_2_	+3	0.770
Crabrolin-9R	FLPLILRKRVTAL-NH_2_	+3	0.761
Crabrolin-TR	FLPRILRKIVRAL-NH_2_	+4	0.671
Crabrolin-FR	RLPRILRKIVRAL-NH_2_	+5	0.455
Crabrolin-AR	RLPRILRKIVRRL-NH_2_	+6	0.354
Crabrolin-PR	RLRRILRKIVRRL-NH_2_	+7	0.221

**Table 2:** The minimum inhibitory concentration (MIC) and minimum bactericidal concentration (MBC) values of crabrolin and its derivatives against tested bacteria. A total of 20 mg/L norfloxacin was treated as a positive control, and phosphate-buffered saline (PBS) was regarded as a negative control.

Peptides	MIC/MBC (µM)					
	*S. aureus* (NCTC 10788)	*E. coli* (ATCC 8739)	MRSA (NCTC 12493)	*K. pneumoniae* (ATCC 43816)	*E. faecium* (NTCC 12697)	*P. aeruginosa* (ATCC 9027)
Crabrolin	2/4	32/128	4/8	64/128	16/64	128/256
Crabrolin-4R	2/4	4/4	4/8	8/8	16/32	16/16
Crabrolin-4K	2/4	4/8	8/16	16/32	32/64	16/32
Crabrolin-9R	256/>512	256/>512	>512/>512	>512/>512	>512/>512	>512/>512
Crabrolin-TR	1/2	2/4	2/2	8/8	4/8	4/8
Crabrolin-FR	2/2	2/4	4/4	8/16	8/16	4/16
Crabrolin-AR	4/8	2/4	4/8	8/16	16/32	4/16
Crabrolin-PR	2/4	2/8	4/8	8/16	8/16	4/16

**Table 3:** The biofilm-inhibiting and eradication activity of peptides against tested bacteria.

Peptides	MBIC/MBEC (µM)			
	MRSA (NCTC 12493)	*K. pneumoniae* (ATCC 43816)	*E. faecium* (NTCC12697)	*P. aeruginosa* (ATCC 9027)
Crabrolin	4/128	>512/>512	32/>512	128/512
Crabrolin-4R	8/64	16/128	16/>512	128/256
Crabrolin-4K	16/128	16/256	16/>512	512/512
Crabrolin-9R	>512/>512	>512/>512	>512/>512	>512/>512
Crabrolin-TR	2/64	8/128	16/>512	8/256
Crabrolin-FR	8/256	8/512	32/>512	8/>512
Crabrolin-AR	8/512	8/>512	64/>512	16/>512
Crabrolin-PR	8/512	8/>512	32/>512	8/>512

**Table 4:** The IC_50_ value (µM) of crabrolin and its derivatives against H838, PC-3, and U251MG cell lines.

Peptides	IC_50_(µM)					
	H838	PC3	U251MG	HCT-116	MCF-7	HaCaT
Crabrolin	27.71	32.13	20.13	8.539	26.49	31.23
Crabrolin-4R	11.40	17.98	25.09	47.02	20.26	35.75
Crabrolin-4K	22.55	20.58	23.51	42.88	23.18	32.57
Crabrolin-9R	2745	353.7	1145	1877	6336	5848
Crabrolin-TR	4.637	3.944	12.40	2.810	3.459	20.85
Crabrolin-FR	31.51	15.87	62.17	30.29	33.85	30.41
Crabrolin-AR	41.31	28.78	52.01	29.90	32.51	34.93
Crabrolin-PR	32.32	22.37	17.70	16.29	12.29	29.53

**Table 5:** The 50% hemolytic concentration (HC_50_), 10% hemolytic concentration (HC_10_), and hemolysis rate at the MIC value of crabrolin and its derivatives.

Peptides	HC_50_ (μM)	HC_10_ (μM)	Hemolysis under the Maximum MIC (%)
Crabrolin	120.654	34.575	53.51
Crabrolin-4R	216.266	62.117	6.18
Crabrolin-4K	217.816	68.628	10.44
Crabrolin-9R	-	-	-
Crabrolin-TR	211.927	36.041	3.76
Crabrolin-FR	-	18.082	5.67
Crabrolin-AR	53230	59.049	6.63
Crabrolin-PR	-	5019	3.09

“-” Represents beyond the statistical scope.

## Data Availability

Not applicable.
